# GRIM-19 Restricts HCV Replication by Attenuating Intracellular Lipid Accumulation

**DOI:** 10.3389/fmicb.2017.00576

**Published:** 2017-04-11

**Authors:** Jung-Hee Kim, Pil S. Sung, Eun B. Lee, Wonhee Hur, Dong J. Park, Eui-Cheol Shin, Marc P. Windisch, Seung K. Yoon

**Affiliations:** ^1^The Catholic University Liver Research Center and WHO Collaborating Center of Viral Hepatitis, The Catholic University of KoreaSeoul, South Korea; ^2^Laboratory of Immunology and Infectious Diseases, Graduate School of Medical Science and Engineering, Korea Advanced Institute of Science and TechnologyDaejeon, South Korea; ^3^Hepatitis Research Laboratory, Discovery Biology Department, Institut Pasteur Korea, Seongnam-siGyeonggi-do, South Korea

**Keywords:** hepatitis C virus, anti-viral host factor, viral replication, lipogenesis, intracellular lipid accumulation

## Abstract

Gene-associated with retinoid-interferon-induced mortality 19 (GRIM-19) targets multiple signaling pathways involved in cell death and growth. However, the role of GRIM-19 in the pathogenesis of hepatitis virus infections remains unexplored. Here, we investigated the restrictive effects of GRIM-19 on the replication of hepatitis C virus (HCV). We found that GRIM-19 protein levels were reduced in HCV-infected Huh7 cells and Huh7 cells harboring HCV replicons. Moreover, ectopically expressed GRIM-19 caused a reduction in both intracellular viral RNA levels and secreted viruses in HCVcc-infected cell cultures. The restrictive effect on HCV replication was restored by treatment with siRNA against GRIM-19. Interestingly, GRIM-19 overexpression did not alter the level of phosphorylated STAT3 or its subcellular distribution. Strikingly, forced expression of GRIM-19 attenuated an increase in intracellular lipid droplets after oleic acid (OA) treatment or HCVcc infection. GRIM-19 overexpression abrogated fatty acid-induced upregulation of sterol regulatory element-binding transcription factor-1 (SREBP-1c), resulting in attenuated expression of its target genes such as fatty acid synthase (FAS) and acetyl CoA carboxylase (ACC). Treatment with OA or overexpression of SREBP-1c in GRIM-19-expressing, HCVcc-infected cells restored HCV replication. Our results suggest that GRIM-19 interferes with HCV replication by attenuating intracellular lipid accumulation and therefore is an anti-viral host factor that could be a promising target for HCV treatment.

## Introduction

After entry into hepatocytes, hepatitis C virus (HCV) becomes uncoated, and the viral genome is translated into a single polyprotein that is co- and post-translationally processed into structural and non-structural proteins (Gale and Foy, 2005; Lindenbach and Rice, 2005). The HCV non-structural proteins, such as NS3 helicase, NS5A, and NS5B RNA-dependent RNA polymerase, assemble as a replicase complex (RC) that is associated with lipid-rich membrane structures (Aizaki et al., 2004; Lindenbach and Rice, 2005). The newly synthesized viral genomes are packaged into viral particles by the structural proteins, including core, E1, and E2. It has been reported that lipid droplets (LDs) are important organelles for the viral packing step in HCV production (Miyanari et al., 2007). Moreover, newly assembled HCV particles are observed in close proximity to LDs, indicating that some steps of virus assembly occur near LDs (Miyanari et al., 2007). The resulting virus is released from the hepatocyte in association with host lipoproteins, and therefore, in the blood, HCV is present as a lipoprotein-coated virus (Meredith et al., 2012). Host lipid architectures and molecules involved in lipid metabolism are closely associated with the HCV lifecycle (Suzuki, 2012). Many studies have shown that lots of host factors participate in HCV infection and play important roles in efficient viral replication and propagation (Li et al., 2009). One such host factor is signal transducer and activator of transcription 3 (STAT3) (McCartney et al., 2013; Kong et al., 2016; Vallianou et al., 2016). It was reported that HCV core interacts with and activates STAT3. The interaction induces expression of STAT3-dependent genes, such as Bcl-XL and cyclin-D1, resulting in cellular transformation (Yoshida et al., 2002). Another study has shown that STAT3 enhances HCV replication through positive regulation of microtubule dynamics (McCartney et al., 2013). More recently, it has been demonstrated that HCV NS4B induces the production of reactive oxygen species (ROS) via the endoplasmic reticulum overload response (EOR)-mediated cancer-related STAT3 pathway (Kong et al., 2016). Furthermore, the roles of cellular regulators of STAT3 such as protein inhibitor of activated STAT (PIAS) and suppressor of cytokine signaling (SOCS3) have been investigated in the context of HCV pathogenesis (El-Saadany et al., 2013; Li Q. et al., 2014; Xu et al., 2014; Aslam et al., 2016; Zhao et al., 2016). However, the function of gene-associated with retinoid-interferon-induced mortality 19 (GRIM-19), which is another cellular inhibitor of STAT3, remains largely unexplored in HCV infection.

GRIM-19 was identified as an interferon (IFN)-β- and retinoic acid (RA)-induced gene with pro-apoptotic properties in breast cancer cell lines (Angell et al., 2000). Studies have demonstrated that GRIM-19 targets multiple signaling pathways and plays a critical role in controlling cell death and growth. Overexpression of GRIM-19 induces cell death, and its suppression or inactivation promotes cell growth (Moreira et al., 2011). Regarding the role of GRIM-19 in cancer development, GRIM-19 expression was severely downregulated in a number of primary renal cell carcinomas (Alchanati et al., 2006), as well as in hepatocellular carcinoma (Liu et al., 2014) and oral squamous cell carcinoma (Li M. et al., 2014). Accordingly, upregulation of GRIM-19 can suppress the growth of specific cancers (Li M. et al., 2014; Liu et al., 2014). These tumor-suppressive activities of GRIM-19 may be attributed to its inhibitory role in the function of STAT3. GRIM-19 was shown to suppress STAT3-induced gene expression via direct interaction with the trans-activation domain (TAD) of STAT3 (Nallar et al., 2008). In this way, binding of GRIM-19 to STAT3 induces changes in the intracellular distribution of STAT3 and renders cells sensitive to cell death (Shulga and Pastorino, 2012).

Interestingly, the function of GRIM-19 was reported to be impeded by viral factors of oncogenic viruses (Kalvakolanu et al., 2010). The viral interferon regulatory factors (vIRFs) from human herpesvirus-8 (HHV-8), implicated in cellular transformation, bind to GRIM-19 and block its ability to induce apoptosis (Seo et al., 2002). Similarly, a non-coding 2.7-kb viral RNA (β2.7) produced by human cytomegalovirus (CMV) enters mitochondria and locks GRIM-19 into Complex-I, rendering it incapable of triggering apoptosis (Reeves et al., 2007).

In this study, we investigated the role of GRIM-19 as a host factor restricting HCV infection. We observed that HCV infection downregulates GRIM-19 at the post-transcriptional level and that GRIM-19 overexpression interferes with HCV replication. Regarding the mechanism for these effects, we found that GRIM-19 decreases intracellular lipid accumulation by regulating the expression of the sterol regulatory element-binding transcription factor-1 (SREBP-1c) gene and its downstream genes. These results suggest that GRIM-19 may be an anti-viral host factor that could be exploited for the development of novel antiviral agents.

## Materials and Methods

### Antibodies and Reagents

Mouse monoclonal anti-GRIM-19 antibody was purchased from Abcam (Cambridge, MA, USA). Mouse monoclonal anti-β-actin and mouse monoclonal anti-flag antibodies were obtained from Sigma–Aldrich (St. Louis, MO, USA). Mouse monoclonal anti-HCV core antibody was purchased from Thermo Scientific (Rockford, IL, USA). Mouse monoclonal anti-HCV NS5A antibody was obtained from Virogen (Watertown, MA, USA). Polyclonal antibodies specific to phospho-STAT3, STAT3 (Ser-705), and acetyl CoA carboxylase (ACC) were purchased from Cell Signaling Technology, Inc. (Danvers, MA, USA). Horseradish peroxidase (HRP)-conjugated anti-mouse, anti-rabbit immunoglobulin G (IgG), mouse monoclonal anti-SREBP-1c, mouse monoclonal anti-fatty acid synthase (FAS), and goat polyclonal anti-stearoyl CoA desaturase (SCD) antibodies were obtained from Santa Cruz Biotechnology (Santa Cruz, CA, USA). Scrambled siRNA and siRNA targeting GRIM-19 were obtained from Santa Cruz Biotechnology.

### Clinical Materials and Ethics Statement

Four liver tissues from patients with chronic HCV infection were obtained during surgical procedures such as cholecystectomy, adrenalectomy, or partial liver resection for intrahepatic duct stones (Seoul St. Mary’s Hospital, Seoul, South Korea). Three out of four patients had cirrhotic liver, and the other patient had chronic hepatitis without cirrhosis. None of them had history of anti-viral treatment. In addition, four liver tissues without viral hepatitis were also obtained during surgical procedures, and they were described in the previous report (Sung et al., 2015). The study conformed to the current ethical principles of the Declaration of Helsinki and was approved by the Institutional Review Board of both Seoul St. Mary’s Hospital and Daejeon St. Mary’s Hospital at the Catholic University of Korea. All patients who provided their tissues completed written informed consents before inclusion in the study. Additionally, their personal identifying information was restricted for analysis purposes and is not available to the public.

### Cell Culture

Huh7 cells were kindly provided by Dr. Jane C. Moores (The Regent of the University of California, Oakland, CA, USA). Dr. Francis Chisari (The Scripps Research Institute, CA) generously provided Huh7.5.1 cells. The cells were cultured in Dulbecco’s modified Eagle’s medium (DMEM; Invitrogen, Carlsbad, CA, USA) supplemented with 10% fetal bovine serum (FBS), 1% antibiotics (100 μg/mL of penicillin, 0.25 μg/mL of streptomycin), and 10 μM HEPES in a humidified incubator at 37°C in 5% CO_2_.

### HCVcc Preparation and Infection

Full-length, infectious HCV RNA of the genotype 2a HCV clone JFH1 was prepared by *in vitro* transcription using a MEGAscript T7 kit (Ambion) and electroporated into Huh7 cells to obtain cell culture-derived HCV (HCVcc) as previously described (Wakita et al., 2005). Huh7 cells were infected with HCVcc at a multiplicity of infection (MOI) of 0.3 by adsorption for 6 h with periodic rocking and then maintained in complete DMEM as previously described (Sun et al., 2012).

### HCV Replicon Systems

An HCV subgenomic replicon (SGR) construct (pSGR-JFH1) and an HCV full-genomic replicon (FGR) construct (pFGR-JFH1) were kindly provided by Dr. Takaji Wakita (National Institute of Infectious Diseases, Tokyo, Japan). The constructs were linearized and then used for *in vitro* transcription as described above. Huh7 cell-derived cell lines containing the HCV SGR or HCV FGR were established by transfection of *in vitro*-transcribed HCV subgenomic or HCV full-genomic RNA, followed by selection with 500 μg/mL G418 sulfate as previously described (Date et al., 2007). The selected cell lines were maintained in complete DMEM containing 500 μg/mL G418 sulfate. HCV genotype-3 replicon cells derived from Huh7.5.1 cells were kindly provided from Dr. Sung Key Jang (Pohang University of Science and Technology, Pohang, Kyungbuk, South Korea).

### Western Blot Analysis

Huh7 cells and Huh7 cells in which HCV replication occurs were lysed with PRO-PREP Protein Extraction Solution (iNtRon BIOTECHNOLGY) containing protease inhibitors. Total protein content was determined using a Bradford protein assay kit (Bio-Rad Laboratories, Hercules, CA, USA). Thirty micrograms of the extracted proteins were subjected to western blot analysis. The analysis was performed as previously described (Choi et al., 2015). The density of each band was analyzed using the Multi Gauge V3.0 program (Fujifilm, Tokyo, Japan).

### Plasmids

pcDNA3_GRIM-19 was constructed to overexpress GRIM-19. The GRIM-19 gene was amplified by PCR with GRIM-19-specific primers (GRIM-19-*Hin*dIII-F, 5′-CCCAAGCTTACCATGGCGGCGTCAAAGGTG-3′ and GRIM-19-*Eco*R I-R, 5′-CGGAATTCTTACGTGTACCACATGAAGCCG-3′) using cDNAs that were reverse transcribed using random primers from RNA extracted from Huh7 cells. The PCR products were cut with *Hin*dIII and *Eco*RI and inserted into a pcDNA3 vector (Invitrogen) in frame. To evaluate the efficiency of transfection with foreign gene-encoding plasmid in Huh7 cells, pEGFP-C1_GRIM-19 was constructed. The GRIM-19 gene was amplified by PCR with GRIM-19-specific primers (GRIM-19- *Bgl*II, GGAAGATCTATGGCGGCGTCAAAGGTGAAG and GRIM-19-*Eco*RI-R) as described above. The PCR products were cut with *Bgl*II and *Eco*RI and inserted into the pEGFP-C1 vector (Clontech Laboratories, Mountain View, CA, USA) in frame. pcDNA3_EGFP was kindly provided by Dr. Sean B. Lee (Tulane University, New Orleans, LA, USA). pcDNA3.1-2xflag-SREBP1c was purchased from Addgene (Cambridge, MA, USA).

### Transient Transfection

To investigate the effects of GRIM-19 on HCV replication and lipogenesis, Huh7 cells or Huh7 cells in which HCV replication occurs were transfected with various plasmids as described above using FUGENE HD (Promega, Madison, WI, USA) according to the manufacturer’s protocol.

### Real-time Quantitative Reverse Transcription-PCR (rqRT-PCR)

The levels of HCV RNA in Huh7 cells infected with HCVcc were evaluated to verify the anti-HCV effects of GRIM-19. Total RNA was extracted with TRIzol reagent (Invitrogen) and purified according to the manufacturer’s recommendations. cDNA was synthesized from 2 μg of total RNA with primers specific for the HCV 5′UTR (HCV-5′UTR-R, 5′-ACCACAAGGCCTTTCGCAACCCAACGCTAC-3′) using ImProm-II reverse transcriptase (Promega). cDNA was then subjected to real-time, quantitative RT-PCR (rqRT-PCR) using primer pairs and a TaqMan probe targeting a region within the HCV 5′UTR as previously described (Kim et al., 2009). rqRT-PCR was performed using a LightCycler 480 Probes Master kit (Roche Applied Science) and a LightCycler 480 system (Roche Applied Science) according to the manufacturer’s instructions. Endogenous mRNA levels of GRIM-19 and genes involved in lipid metabolism were also assessed using the LightCycler 480 Probes Master kit and the LightCycler 480 system with gene-specific primers and fluorescent probes recommended by Roche Universal Probe Library Design Center. The thermal conditions were designed using the Roche Universal Probe Library’s thermocycling conditions following the manufacturer’s instructions. Human β-actin was used as a reference gene. All fluorescence data were analyzed using LightCycler 4.0 software (Roche Applied Science), and *C*_t_ results were exported to Excel spreadsheets. The comparative *C*_t_ method was used for relative quantification and normalization.

### Dual-luciferase Assay

Changes in HCV-internal ribosome entry site (IRES) activity were confirmed by a dual-luciferase assay. A dual-luciferase reporter construct was kindly provided by Dr. Jong-Won Oh (Yonsei University, Seoul, South Korea). It contains a CMV promoter-controlled *Renilla* luciferase reporter gene followed by the HCV IRES-controlled firefly luciferase reporter gene. Huh7 cells infected with HCVcc were cotransfected with the dual-luciferase reporter construct and pcDNA3_GRIM-19 using fuGENE HD. At 48 h post-transfection, dual-luciferase assays were performed with a Dual-Luciferase Reporter Assay System (Promega) according to the manufacturer’s instructions.

### Subcellular Fractionation

Huh7 cells infected with HCVcc were transfected with pcDNA3 or pcDNA3_GRIM-19. After 48 h, the cells were subjected to subcellular fractionation into nuclear and cytoplasmic fractions using an NE-PER kit (Pierce, Rockford, IL, USA) according to the manufacturer’s recommendations.

### Reverse Transcription-polymerase Chain Reaction (RT-PCR)

The mRNA levels of *bcl2* and *mmp2* were evaluated using RT-PCR. Total RNA extraction and cDNA synthesis using random primers were performed as described above. Gene amplification was performed with GoTaq Polymerase (Promega) and specific primer pairs for *bcl2* (bcl2-F, 5′-TCCCTCGCTGCACAAATACTC-3′, and bcl2-R, 5′-TTCTGCCCCTGCCAAATCT-3′) and *mmp2* (mmp2-F, 5′-CCACTGCCTTCGATACAC-3′, and mmp2-R, 5′-GAGCCACTCTCTGGAATCTTAAA-3′). The PCR program ran as follows: 10 min at 94°C; 30 cycles of 94°C for 30 s, 55°C for 30 s, and 72°C for 45 s; followed by a final 10 min incubation at 72°C. The amplified products were separated on 1.5% agarose gels containing 0.5 mg/mL ethidium bromide. The nucleic acids were visualized under UV light using a Gel-Doc CQ system (Bio-Rad, Vienna, Austria), and the band densities of each gene were analyzed using the Multi Gauge V3.0 program with β-actin serving as a loading control.

### Apoptosis Assays

Apoptosis was detected with Annexin V/propidium iodide (PI) staining (BD BioSciences) according to the manufacturer’s instructions. In total, 10, 000 cells were counted by flow cytometry using a fluorescence-activated cell sorter (FACS, Becton-Dickinson, San Jose, CA, USA). The resulting data were analyzed using Summit 5.2 software (Beckman Coulter Inc., Miami, FL, USA).

### Intracellular Lipid Droplet Quantification

Huh7 cells and HCVcc-infected Huh7 cells were treated with 100 μM oleic acid (OA) in serum-free DMEM containing 1% BSA at 24 h post-transfection with pcDNA3_GRIM-19 or pEGFP-C1_GRIM-19. Twenty-four hours later, the cells were subjected to Nile Red staining to evaluate the changes in intracellular lipid content. The cells were washed with ice-cold phosphate-buffered saline (PBS) and fixed with 4% paraformaldehyde for 5 min at room temperature. After being washed with PBS again, the cells were stained with Nile Red (0.5 μg/mL) and 4′,6-diamidino-2-phenyl-indole (DAPI, 1 μg/mL) (Sigma–Aldrich). After staining, intracellular LDs were quantified by measuring density of fluorescence with a microplate reader (Molecular Devices, Sunnyvale, CA, USA), and the results were normalized to the cellular DAPI content (Hur et al., 2012). The distribution of lipid in cells was observed under an LSM 510 inverted laser-scanning confocal microscope (Carl Zeiss, Jena, Germany).

### Immunofluorescence Staining

Huh7 cells and HCVcc-infected Huh7 cells were fixed with 4% paraformaldehyde for 30 min, and permeabilized with PBS containing 0.2% Triton X-100 for 30 min at room temperature. After washing three times with PBS, the cells were treated with a blocking solution (PBS containing 1% BSA, 0.1% gelatin, and 5% goat serum) for 30 min at room temperature, incubated with primary antibody overnight at 4°C, and washed five times with PBS containing 1% BSA and 0.1% gelatin. The cells were further incubated with secondary antibodies (Molecular Probes, Eugene, OR, USA) for 2 h and washed five times with PBS. Nuclei were visualized using (DAPI) in PBS for 10 min. Stained slides were observed under an LSM 510 inverted laser-scanning confocal microscope (Carl Zeiss).

### Statistical Analysis

All data are representative of a minimum of three independent experiments. The data are expressed as the mean ± SD or ±SEM. For comparison of multiple groups, one-way analysis of variance (ANOVA) with Tukey’s *post hoc* test was used to define statistically significant differences among groups. For statistical comparisons between two groups, Student’s *t*-test was used. The statistical significance of differences between groups is expressed by an asterisk (^∗^*P* < 0.05, ^∗∗^*P* < 0.01, ^∗∗∗^*P* < 0.001).

## Results

### GRIM-19 Protein Levels Are Downregulated in HCV-infected Cells

**FIGURE 1:**
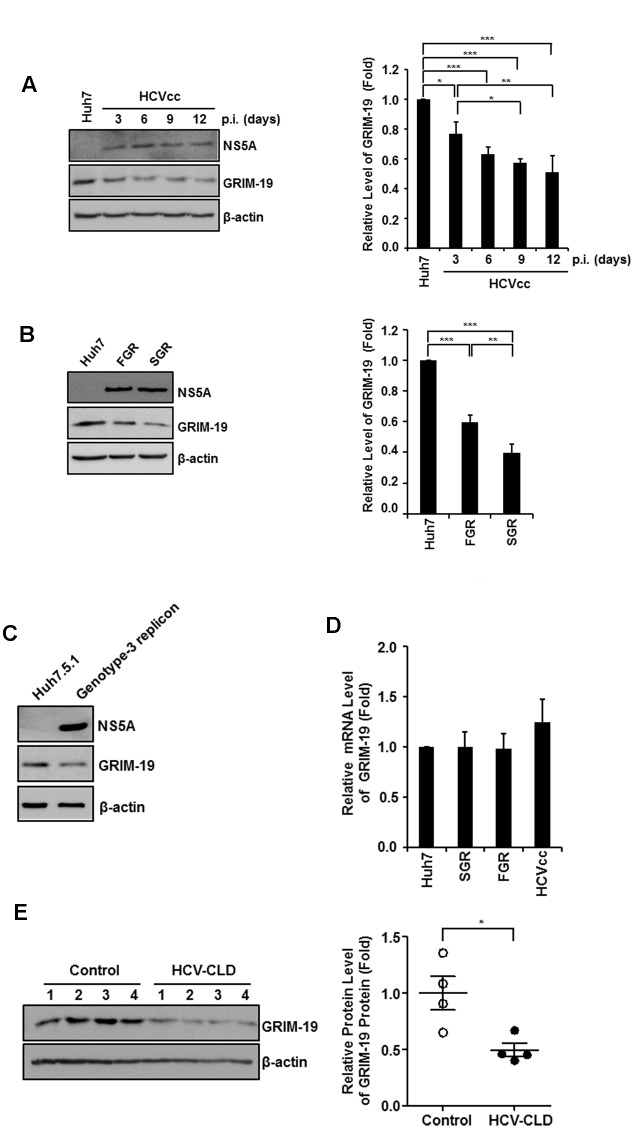
**Hepatitis C virus (HCV) infection and viral replication reduced GRIM-19 expression.** **(A,B)** Protein levels of GRIM-19 were evaluated using western blot analysis in Huh7 cells infected with HCVcc at days 3, 6, 9, and 12 post-infection **(A)**, FGR cells **(B)**, and SGR cells **(B)**. The relative protein expression was normalized to β-actin as a reference. **(C)** The endogenous GRIM-19 level was assessed by western blot analysis in Huh7.5.1 cells or Huh7.5.1-derived HCV genotype 3 full genomic replicon cells. The relative protein expression was normalized to β-actin as a reference. **(D)** Relative levels of endogenous GRIM-19 mRNA in SGR cells, FGR cells, and HCVcc-infected Huh7 cells compared to that in Huh7 cells. β-actin was used as a reference gene. **(E)** Protein levels of GRIM-19 in liver tissues from patients with chronic liver diseases (CLD) caused by persistent HCV infection (*n* = 4) were analyzed by western blot analysis. Tissue lysates from liver without viral hepatitis were used as a control (normal; *n* = 4). β-actin was used as a loading control. The values of the GRIM-19 protein levels were expressed relative to the level in control tissues (right). All data represent the mean ± SEM (*n* = 3). ^∗^*P* < 0.05, ^∗∗^*P* < 0.01, ^∗∗∗^*P* < 0.001 compared to control.

First, we examined the expression of GRIM-19 protein in Huh7 cells infected with genotype 2a HCVcc. As shown in **Figure 1A**, GRIM-19 expression decreased in Huh7 cells infected with HCVcc. On the 3rd day of HCV infection, protein level of GRIM-19 was downregulated by approximately 20%. Moreover, GRIM-19 expression was decreased by approximately 50% on the 12th day of HCV infection. To confirm the downregulation of GRIM-19 in HCV-replicating cells, we assessed GRIM-19 expression in genotype 2a HCV SGR cells and FGR cells. In these cells, the GRIM-19 protein level was lower than that in Huh7 control cells (**Figure 1B**). Furthermore, in the genotype 3 HCV FGR cell line derived from Huh7.5.1 cells, the expression level of GRIM-19 was lower compared to that in parental Huh7.5.1 cells (**Figure 1C**). Interestingly, GRIM-19 mRNA levels were not altered in the cells with active HCV replication (**Figure 1D**). Next, we determined the protein levels of GRIM-19 in liver tissues from patients with chronic HCV infection. Compared to the liver tissues without viral hepatitis, HCV-infected livers expressed markedly lower levels of GRIM-19 protein (**Figure 1E**). These results suggest that HCV infection causes downregulation of GRIM-19 at the post-transcriptional level.

### Ectopically Expressed GRIM-19 Reduces HCV RNA Replication

**FIGURE 2:**
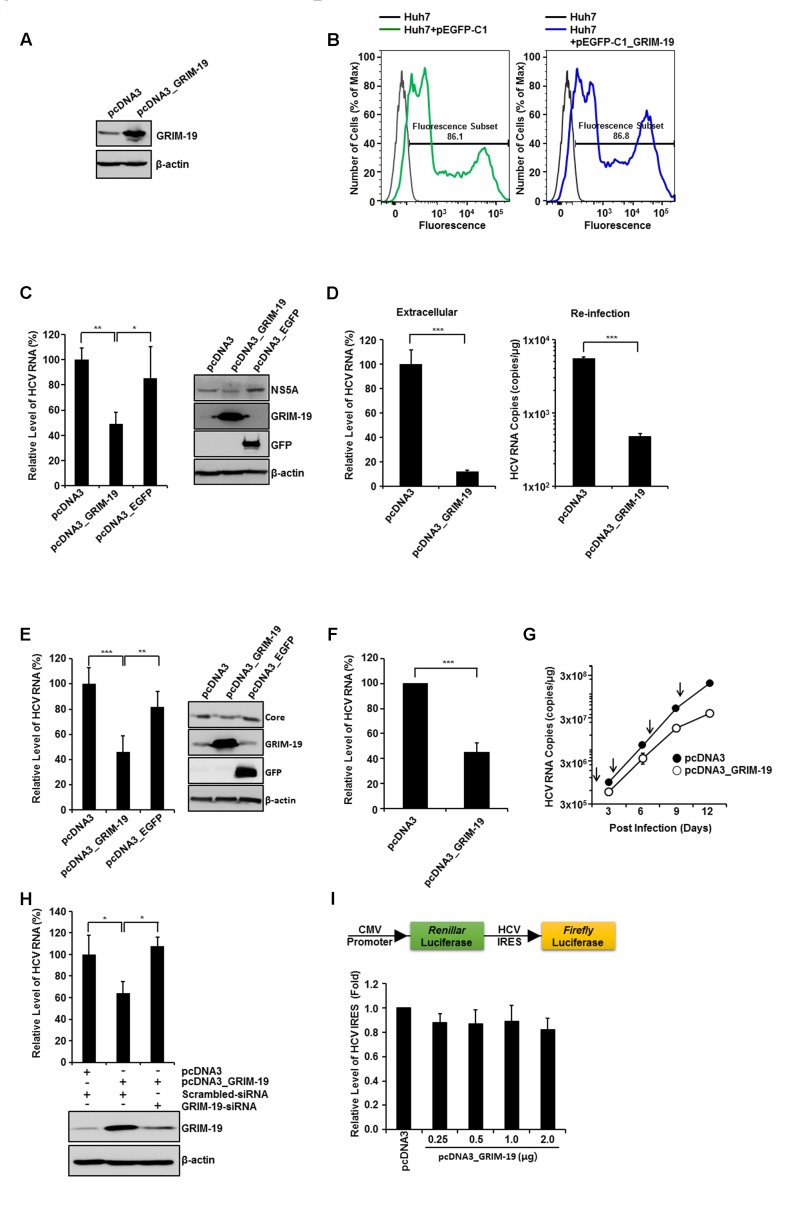
**Anti-HCV activity of ectopically expressed GRIM-19.** **(A)** Overexpression of GRIM-19 was induced by transfection with pcDNA3_GRIM-19 in Huh7 cells. At 48 h post-transfection, GRIM-19 overexpression was detected by western blot analysis. **(B)** Huh7 cells were transfected with pEGFP-C1 or pEGFP-C1-GRIM-19. After 48 h, the cells were subjected to flow cytometry to evaluate the transfection efficiency. **(C)** HCVcc-infected Huh7 cells at day 9 post-infection were transfected with pcDNA3, pcDNA3_GRIM-19 or pcDNA3_EGFP. After 48 h, the intracellular levels of HCV RNA were evaluated by rqRT PCR (left), and the protein level of HCV NS5A was detected by western blot analysis (right). The values of the HCV RNA levels were expressed relative to the level in cells transfected with pcDNA3. **(D)** At day 9 post-infection, HCVcc-infected Huh7 cells were transfected with pcDNA3 or pcDNA3_GRIM-19. After 48 h, the culture media was used to evaluate the extracellular level of HCV RNA (left) as well as for re-infection. After another 48 h, the HCV RNA levels in re-infected Huh7 cells were evaluated by rqRT PCR. **(E)** Levels of HCV RNA and HCV core protein in FGR cells were analyzed after transfection with pcDNA3, pcDNA3_GRIM-19 or pcDNA3_EGFP as in **(C)**. **(F)** The values of the HCV RNA levels are analyzed in SGR cells transfected with pcDNA3 or pcDNA3_GRIM-19 as in **(C)**. **(G)** To examine the inhibitory effect of repeated transfection with pcDNA3_GRIM-19, Huh7 cells were infected with HCVcc at an MOI of 0.3. After 24 h, cells were transfected with pcDNA3 or pcDNA3_GRIM-19. The cells were maintained at 3-day intervals and transfected with pcDNA3 or pcDNA3_GRIM-19 repeatedly. At 3, 6, 9, and 12 days post-infection, cells were harvested, and HCV RNA levels were evaluated. **(H)** At day 9 post-infection, HCVcc-infected Huh7 cells were transfected with pcDNA3 or pcDNA3_GRIM-19. After 24 h, the cells were transfected with scrambled siRNA or GRIM-19-siRNA. Changes in GRIM-19 levels were assessed by western blot analysis 48 h post-transfection with siRNAs (bottom). In addition, HCV RNA levels were evaluated by rqRT-PCR (top). **(I)** HCV-IRES activity in Huh7 transfected with pcDNA3 or pcDNA3_GRIM-19. The data represent the means ± SEM (*n* = 3). ^∗^*P* < 0.05, ^∗∗^*P* < 0.01, ^∗∗∗^*P* < 0.001 compared to control.

To investigate the roles of GRIM-19 in the HCV viral life cycle, we overexpressed GRIM-19 in HCVcc-infected or HCV replicon cells (**Figure 2**). GRIM-19 was ectopically expressed in the cells via transient transfection as described in section “Materials and Methods”. Before evaluation of the effect of GRIM-19 overexpression on HCV replication, the transfection efficiency of Huh7 cells was checked using flow cytometry after transfection with enhanced green fluorescent protein (EGFP)-fused GRIM-19-encoding plasmids. As shown in **Figure 2B**, GFP fluorescence was detected in a high percentage of Huh7 cells transfected with EGFP-fused GRIM-19-encoding plasmids, even though the density of fluorescence among the cells was different. In the same transient transfection conditions, GRIM-19 was overexpressed in HCVcc-infected Huh7 cells. When Huh7 cells were infected with HCVcc at an MOI of 0.3, the level of intracellular HCV RNA was gradually increased until the 12th day after infection (**Figure 2G**). The level of intracellular HCV RNA on the 15th day was comparable to that on the 12th day (data not shown). These results indicate that the ratio of Huh7 cells infected with HCVcc to uninfected cells reached the highest level on approximately the 12th day after HCVcc infection at an MOI of 0.3. Therefore, to examine the effect of GRIM-19 overexpression on HCV replication, Huh7 cells infected with HCVcc were seeded on the 9th day post-infection, and the next day, the cells were transfected with GRIM-19 encoding plasmid. After 48 h, the levels of intracellular HCV RNA and protein were evaluated. As shown in **Figure 2C**, GRIM-19 overexpression resulted in an approximately 50% decrease in the levels of intracellular HCV RNA in Huh7 cells infected with HCVcc. Moreover, transient transfection with GRIM-19-encoding plasmid also reduced the protein level of HCV NS5A (**Figure 2C**, right). Ectopically expressed EGFP did not have an effect on the levels of either intracellular HCV RNA or HCV NS5A protein (**Figure 2C**). Interestingly, GRIM-19 transfected, HCVcc-infected cells secreted a much lower number of viral particles (**Figure 2D**, left). When Huh7 cells were re-infected with culture supernatants obtained from HCVcc-infected, GRIM-19-transfected cells, the levels of intracellular HCV RNA were lower (**Figure 2D**, right). Anti-HCV activity of GRIM-19 was also confirmed in FGR cells and SGR cells. As expected, GRIM-19 overexpression reduced the HCV RNA level to less than 50% in FGR cells and SGR cells (**Figures 2E** right, **F**). Additionally, ectopically expressed GRIM-19 reduced the protein level of HCV core in FGR cells (**Figure 2E**, right). Moreover, in the cells, EGFP overexpression did not have an effect on the levels of either intracellular HCV RNA or HCV core protein (**Figure 2E**). Furthermore, repeated transfection of Huh7 cells infected with HCVcc with GRIM-19 encoding plasmid resulted in additive inhibitory effects on HCV RNA replication (**Figure 2G**). In the first round of transfection with GRIM-19, the level of HCV RNA was 59% that of pcDNA3-transfected cells. After the fourth round of GRIM-19 transfection, the level of HCV RNA was 19% that of vehicle-transfected, HCVcc-infected Huh-7 cells. To confirm the suppressive function of GRIM-19 on HCV replication, we examined whether the inhibitory effect of GRIM-19 on HCV replication could be abolished by siRNA against GRIM-19. In HCVcc-infected, GRIM-19-overexpressing Huh7 cells, transfection with GRIM-19 siRNA abrogated the suppressive effect of GRIM-19 on HCV replication (**Figure 2H**). Collectively, these results suggest that GRIM-19 overexpression inhibits HCV.

### Viral Internal Ribosome Entry Site (IRES)-Mediated Translation of the HCV Genome Is Not Altered by GRIM-19

Next, we investigated whether GRIM-19 overexpression had an effect on HCV-IRES activity. Huh7 cells were transfected with GRIM-19-encoding plasmid and a dual-luciferase reporter construct, allowing cap-dependent expression of *Renilla* luciferase and HCV IRES-dependent translation of firefly luciferase. As shown in **Figure 2I**, forced expression of GRIM-19 did not alter HCV IRES activity. These results suggest that the suppressive effect of GRIM-19 on HCV RNA replication is not caused by alteration of HCV-IRES activity.

### GRIM-19 Overexpression Does Not Alter the Subcellular Localization or the Transcriptional Activity of Phosphorylated STAT3 in HCVcc-infected Cells

**FIGURE 3:**
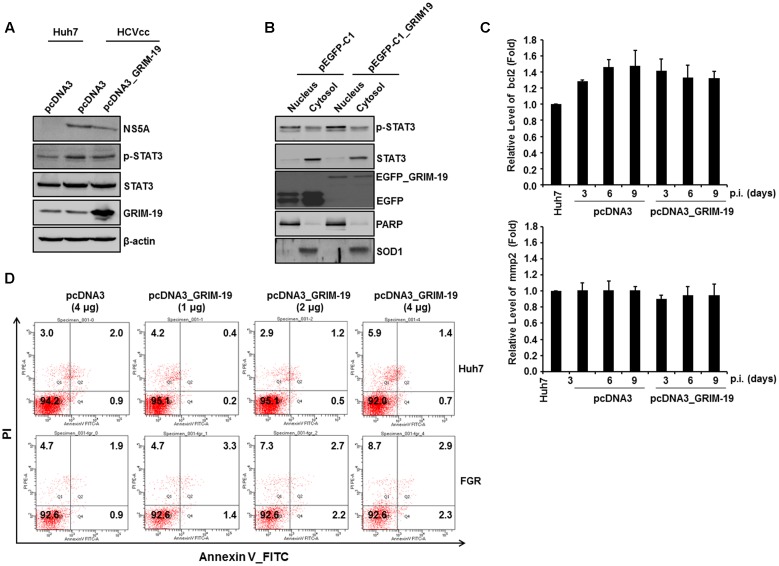
**GRIM-19 overexpression did not alter STAT3 transcriptional activity.** **(A)** The expression and phosphorylation of STAT3 after transfection with pcDNA3 or pcDNA3_GRIM-19 were evaluated by western blot analysis. β-actin was used as an internal control for loading. **(B)** To evaluate subcellular localization of phospho-STAT3, STAT3, and GRIM-19, HCVcc-infected Huh7 cells were transfected with pEGFP-C1 or pEGFP-C1_GRIM-19. After 48 h, the cells were subjected to subcellular fractionation followed by western blot analysis. PARP and SOD1 were used as markers for the nucleus and cytosol. **(C)** The mRNA levels of *bcl2* and *mmp2* were assessed by RT-PCR with a gene-specific primer set. The mRNA expression was normalized to β-actin as a reference and expressed relative to the density of Huh7 cells. Values represent means ± SD. **(D)** Examination of the induction of apoptosis by overexpression of GRIM-19 in Huh7 cells or FGR cells. The cells were transfected with pcDNA3 or pcDNA3_GRIM-19. After 48 h, apoptosis was determined by a Annexin V/PI staining.

As described above, one of the host factors that interact with HCV proteins is STAT3. Because GRIM-19 is known to interact with phosphorylated STAT3 and transport it out of the nucleus (Shulga and Pastorino, 2012), we investigated whether GRIM-19 overexpression has an effect on STAT3 activation in HCVcc-infected Huh7 cells. First, we aimed to confirm the amount and subcellular localization of phosphorylated STAT3 in Huh7 cells infected with HCVcc after transfection with a GRIM-19-encoding plasmid. The level of phosphorylated STAT3 increased after infection with HCVcc in Huh7 cells (**Figure 3A**); however, overexpression of GRIM-19 did not reduce the increased phosphorylation of STAT3 (**Figure 3A**). Furthermore, GRIM-19 overexpression did not induce translocation of phosphorylated STAT3 (**Figure 3B**). Follow-up experiments showed that the expression levels of *bcl2* and *mmp2*, which are induced by transcriptional activation of STAT3, were not altered by overexpression of GRIM-19 (**Figure 3C**). Furthermore, GRIM19 overexpression did not induce sufficient apoptosis to show anti-HCV activity in HCV-replicating Huh7 cells (**Figure 3D**). These results suggest that the inhibitory effect of GRIM-19 on HCV replication is not associated with altered STAT3 activation in HCV-infected cells.

### GRIM-19 Attenuates Intracellular Lipid Accumulation

**FIGURE 4:**
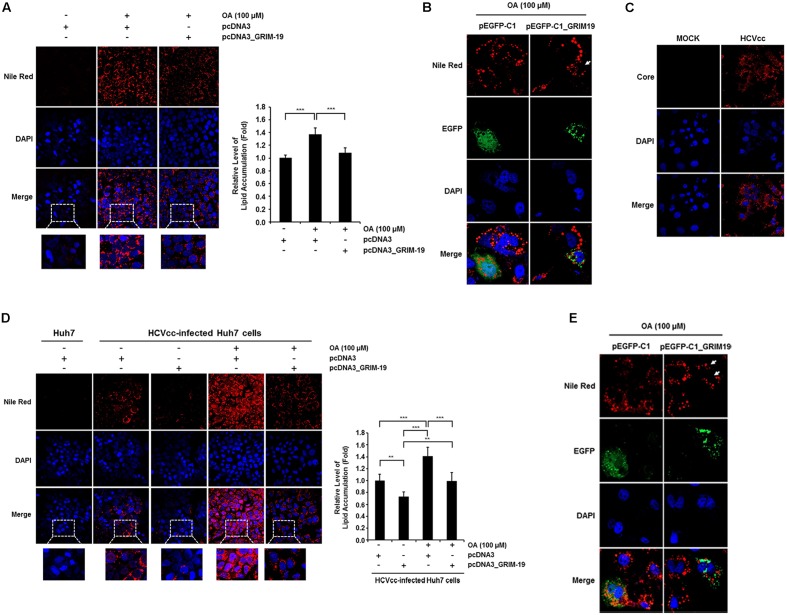
**GRIM-19 overexpression inhibited lipid accumulation.** **(A)** Huh7 cells were transfected with pcDNA3 or pcDNA3_GRIM-19. After 24 h, cells were treated with 100 μM OA. After an additional 24 h, intracellular lipid accumulation was visualized by Nile Red staining and observed under a confocal microscope (left). Red, lipid droplets (LDs); blue, DAPI; original magnification 200×. Fluorescence densities of Nile Red were quantified using a microplate reader and normalized to the cellular DAPI content. The values of intracellular lipid level were expressed relative to the level in the cells transfected with pcDNA3 and without OA treatment (right). The data represent the mean ± SEM (*n* = 3). ^∗∗^*P* < 0.01, ^∗∗∗^*P* < 0.001 compared to control. **(B)** Huh7 cells were transfected with pEGFP-C1 or pEGFP-C1-GRIM-19. After 24 h, cells were treated with 100 μM OA. After an additional 24 h, intracellular lipid accumulation was assessed by Nile Red staining. Red, LDs; Green, EGFP or EGFP-fused GRIM-19; blue, DAPI; original magnification 800×. **(C)** HCV core was immunostained in Huh7 cells infected with HCVcc at day 9 post-infection. Red, HCV core; blue, DAPI; original magnification 400×. **(D)** Changes of intracellular lipid accumulation by GRIM-19 overexpression was assessed in Huh7 cells infected with HCVcc at day 9 post-infection as in **(A)**. **(E)** Smaller size and number of LDs in GRIM-19 overexpressing cells were visualized in HCVcc infected Huh7 cells as in **(B).**

The level of HCV RNA was significantly downregulated by transfection with GRIM-19-encoding plasmids in SGR cells (**Figure 2F**). This suggests that the anti-viral function of GRIM-19 may be closely associated with HCV replication or viral RC formation. As shown in **Figure 2I**, GRIM-19 overexpression does not restrict viral translation. In addition, the dependence of HCV replication and viral RC formation on intracellular lipid accumulation is well known (Kapadia and Chisari, 2005; Mankouri et al., 2010; Pisonero-Vaquero et al., 2014; Akil et al., 2016). Therefore, we further investigated whether GRIM-19 overexpression affects intracellular lipid accumulation. Huh7 cells and HCVcc-infected Huh7 cells were transfected with GRIM-19-encoding plasmids. In both types of cells, treatment with OA increased intracellular lipid levels by up to approximately 140% (**Figures 4A,D**). However, in cells overexpressing GRIM-19, the levels of intracellular lipid accumulation after treatment with OA were comparable to that of untreated cells (**Figures 4A,D**). Moreover, the number and size of LDs after OA treatment were smaller in the cells transfected with EGFP-fused GRIM-19-encoding plasmids compared to that in untransfected cells (**Figures 4B,E**). Furthermore, as shown in **Figure 4C**, HCV core protein was detected in most Huh7 cells infected with HCVcc on the 9th day post-infection, and in the cells, the amount of intracellular lipids increased without OA treatment, as previously reported (**Figure 4D**) (McRae et al., 2015; Akil et al., 2016). Additionally, overexpression of GRIM-19 significantly reduced the level of intracellular lipid accumulation caused by HCV infection (**Figure 4D**). These results may indicate that GRIM-19 can ameliorate intracellular lipid accumulation in hepatocytes.

### GRIM-19 Overexpression Downregulates the Expression Levels of SREBP1-c and Its Target Genes

**FIGURE 5:**
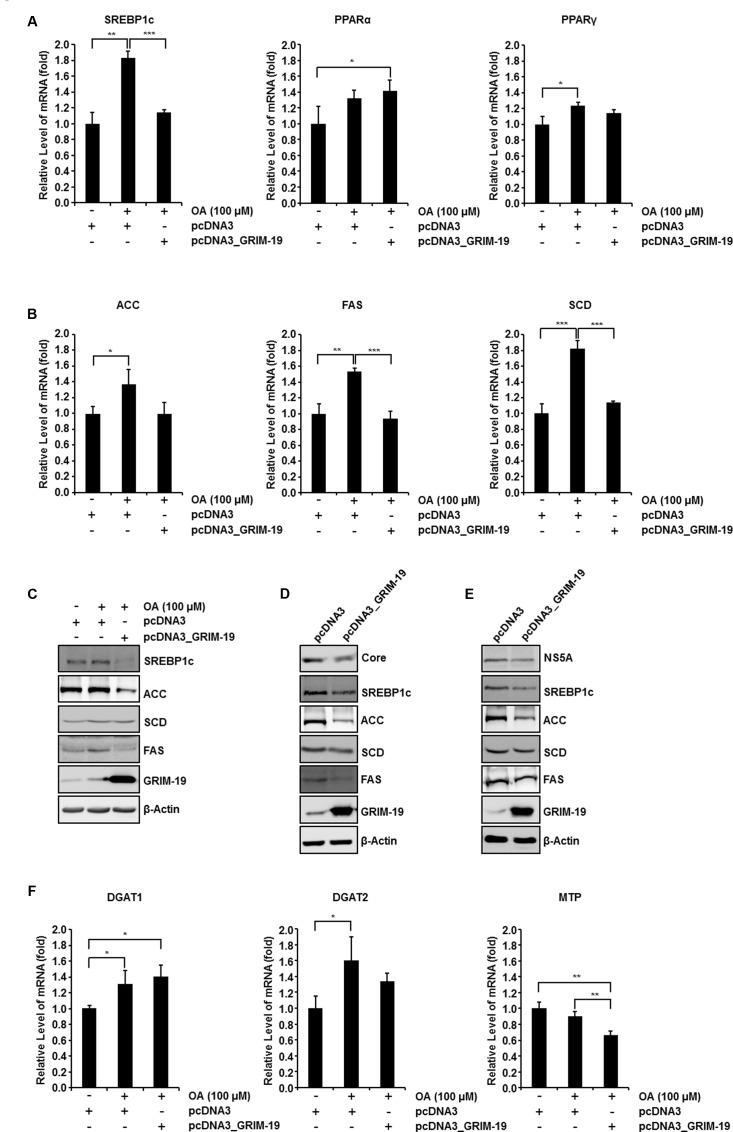
**Effect of GRIM-19 overexpression on the expression levels of genes involved in lipid metabolism.** **(A)** Examination of the mRNA levels of three transcription factors that regulate the intracellular lipid level in Huh7 cells treated with OA or transfected with pcDNA3 or pcDNA3_GRIM-19. The mRNA expression was normalized to β-actin as a reference and the values of mRNA level were expressed relative to the level in cells transfected with pcDNA3 and without OA treatment. **(B)** mRNA levels of target genes of SREBP-1c were analyzed as in **(A). (C)** Protein levels of SREBP-1c and its target genes in Huh7 cells treated as in **(A)** were analyzed using western blot analysis. β-actin was used as an internal control for loading. **(D,E)** Protein levels of SREBP-1c and its target genes after transfection with pcDNA3 or pcDNA3_GRIM-19 in FGR cells **(D)** and Huh7 cells infected with HCVcc at day 9 post-infection **(E)**. β-actin was used as an internal control for loading. **(F)** mRNA levels of DGAT1, DGAT-2, and MTP were analyzed as in **(A)**. The data represent the mean ± SEM (*n* = 3). ^∗^*P* < 0.05, ^∗∗^*P* < 0.01, ^∗∗∗^*P* < 0.001 compared to control.

Next, we investigated the mRNA level of transcription factors involved in regulating the intracellular lipid level after transfection with GRIM-19-encoding plasmids. We examined the expression of the following transcription factors: (i) SREBP-1c, known to induce *de novo* lipogenesis to generate free fatty acid (FFA) (Sanders and Griffin, 2016); (ii) peroxisome proliferator-activated receptor α (PPARα), required for mitochondrial, peroxisomal, and microsomal FFA oxidation (Memon et al., 2000); and (iii) peroxisome proliferator-activated receptor γ (PPARγ), which is known to contribute to FFA uptake (Ahmadian et al., 2013). Among these three transcription factors, only SREBP-1c expression levels were significantly upregulated in Huh7 cells treated with 100 μM OA and downregulated by transfection with GRIM-19-encoding plasmids before OA treatment (**Figures 5A** left, **C**). Interestingly, the mRNA levels of SREBP-1c target genes involved in triglyceride biosynthesis such as FAS, SCD, and ACC were upregulated following treatment with OA and downregulated as a result of GRIM-19 overexpression induced prior to OA treatment (**Figure 5B**). Moreover, increased ACC and FAS protein levels resulting from OA treatment were downregulated by ectopically expressed GRIM-19 (**Figure 5C**). In both FGR cells and Huh7 cells infected with HCVcc, the expression levels of SREBP-1c, FAS, and ACC were remarkably downregulated by GRIM-19 overexpression (**Figures 5D,E**). Unexpectedly, the protein levels of SCD were not significantly altered by GRIM-19 overexpression in the cells (**Figures 5C–E**). Next, we further determined the effect of GRIM-19 on the expression levels of other enzymes involved in lipid metabolism. Interestingly, the expression levels of diacylglycerol acyltransferase-1 (DGAT-1) and diacylglycerol acyltransferase-2 (DGAT-2), which catalyze the final step in triglyceride biosynthesis, were not significantly affected by GRIM-19 overexpression (**Figure 5F**). Likewise, microsomal triglyceride transfer protein (MTP), which is involved in the assembly/secretion of very low density lipoproteins (VLDL), was not upregulated by OA treatment even though overexpression of GRIM-19 decreased the MTP mRNA level (**Figure 5F**). Taken together, these results demonstrate that GRIM-19 may ameliorate intracellular lipid accumulation by regulating the expression of SREBP-1c and its target genes.

### GRIM-19 Restricts HCV Replication through Downregulation of SREBP-1c

**FIGURE 6:**
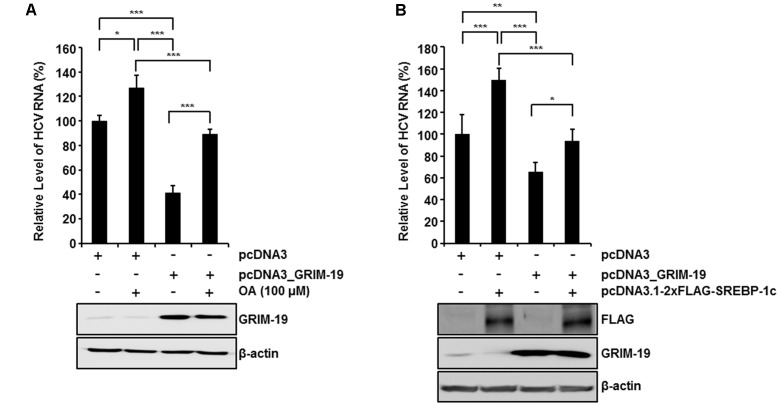
**Restoration of GRIM-19 inhibition of HCV replication.** At day 9 post-infection, HCVcc-infected Huh7 cells were transfected with pcDNA3 or pcDNA3_GRIM-19. After 24 h, the cells were treated with OA **(A)** or transfected with SREBP-1c-overexpressing plasmids **(B)**. After an additional 48 h, intracellular levels of HCV RNA were evaluated by rqRT PCR (top), and the overexpressed protein levels was detected by western blot analysis (bottom). The values of the HCV RNA levels were expressed relative to the level in cells transfected with pcDNA3. The data represent the mean ± SEM (*n* = 3). ^∗^*P* < 0.05, ^∗∗^*P* < 0.01, ^∗∗∗^*P* < 0.001 compared to control.

To confirm the suppressive effect of GRIM-19 on intracellular lipid accumulation, we examined whether the restrictive effect of GRIM-19 on HCV replication could be abolished by OA treatment or normalization of SREBP-1c expression. As shown in **Figure 6A**, OA treatment in the absence of GRIM-19 transfection increased the HCV RNA titer in HCVcc-infected Huh7 cells. Moreover, in HCVcc-infected, GRIM-19-overexpressing Huh7 cells, downregulation of HCV RNA was restored by OA treatment (**Figure 6A**). In the same manner, SREBP-1c overexpression increased the level of HCV RNA and abolished the restrictive effect of GRIM-19 on HCV replication (**Figure 6B**). These results suggest that the inhibitory effect of GRIM-19 overexpression on HCV replication is mediated by downregulation of SREBP-1c.

## Discussion

**FIGURE 7:**
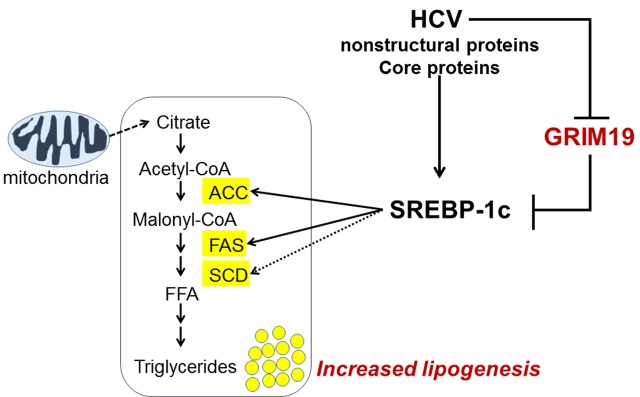
**A proposed model of the mechanism underlying the anti-HCV activity of GRIM-19.** After HCV infection, lipid biosynthesis is upregulated via SREBP-1c activation. SREBP-1c is a transcription factor that enhances the expression of genes involved in fatty acid biosynthesis such as ACC, FAS, and SCD, causing increased levels of triglycerides that help create an appropriate microenvironment for persistent HCV infection. Conversely, GRIM-19 function is downregulated through the reduction of its protein levels in HCV-replicating cells. However, restoration of GRIM-19 expression can disrupt this suitable microenvironment by downregulating SREBP-1c expression.

In this study, we uncovered a new biological function of GRIM-19 in lipid metabolism, as summarized in **Figure 7**. HCV replication caused the GRIM19 protein level to decrease. However, restoration of the downregulated level of GRIM-19 by transient transfection with a GRIM-19-encoding plasmid restricted HCV replication. Interestingly, GRIM-19 overexpression downregulated the expression of SREBP-1c and its target genes, resulting in abrogation of the intracellular lipid accumulation induced by HCV replication. These results suggest that GRIM-19 can be thought of as a host factor that restricts HCV replication.

It is known that HCV exploits host lipid architectures and molecules involved in lipid metabolism for its efficient replication and propagation (Miyanari et al., 2007; Negro and Sanyal, 2009; Syed et al., 2010). For example, HCV genome replication, in common with other positive-strand RNA viruses, occurs within a “membranous web” derived from intracellular vesicles (Egger et al., 2002; Miyanari et al., 2007). Additionally, it has been reported that LDs act as a platform for HCV replication and assembly (Miyanari et al., 2007). HCV core protein recruits HCV RNA, non-structural proteins, and replication complexes to LD-associated membranes. Thus, this recruitment is critical for infectious virus particle production (Miyanari et al., 2007). For these reasons, HCV induces intracellular lipid accumulation to optimize the cellular environment for persistent infection (Kapadia and Chisari, 2005; Mankouri et al., 2010; Akil et al., 2016). As one of the strategies to increase intracellular lipids, HCV activates SREBPs (Waris et al., 2007; Negro and Sanyal, 2009; Syed et al., 2010). Recent research has demonstrated that HCV NS4B, NS5A, and core protein may activate SREBP-1c and its target genes, resulting in enhanced fatty acid biosynthesis (Park et al., 2009; Syed et al., 2010; Xiang et al., 2010; Garcia-Mediavilla et al., 2012). Moreover, inhibiting or silencing SREBP by treatment with 25-hydroxycholesterol inhibits HCV replication (Su et al., 2002; Yang et al., 2008; Li et al., 2013). In the present study, we showed that GRIM-19 overexpression impeded the lipid accumulation induced by OA treatment and HCV infection through downregulation of SREBP-1c expression. Furthermore, the expression of SREBP-1c target genes, such as ACC and FAS but not SCD was markedly downregulated by GRIM-19 overexpression. Luyimbazi et al. (2010) demonstrated that translation of SCD is regulated by eukaryotic initiation factor 4E (eIF4E), suggesting that mTOR may regulate SCD through the mTOR/4E-BP1/eIF4E axis. More recently, it was reported that eIF4E could be activated in HCV-replicating cells for efficient viral translation (George et al., 2012; Licursi et al., 2012; Panda et al., 2014). Based on these reports, we speculate that eIF4E activation by HCV could be one of the reasons that mRNA levels of SCD are decreased by GRIM-19 overexpression but the protein levels of SCD are not affected under the same conditions. However, future investigations are needed to understand the exact mechanisms of a sustained level of SCD protein after transfection with GRIM-19-encoding plasmids. Despite the fact that SCD expression was not modulated by GRIM-19 overexpression, these findings suggest that GRIM-19 may abrogate intracellular lipid accumulation by inhibiting SREBP-1c activation and that these inhibitory effects could restrict HCV replication.

Signal transducer and activator of transcription 3, a major oncogenic transcription factor involved in cancer development and progression, is regulated by GRIM19 in some tumor cell lines (Alchanati et al., 2006; Kalakonda et al., 2007), but little is known regarding their relationships in viral infections. For this reason, we explored a specific functional link between GRIM19 and STAT3 in HCV infection. However, our results showed that the inhibitory effect of GRIM-19 on HCV replication was not associated with altered STAT3 activation in HCV-infected cells. Moreover, the precise mechanism by which GRIM-19 downregulates SREBP-1c gene expression remains unclear. Hence, further investigation is warranted concerning the functional links among GRIM-19, STAT3, and SREBP-1c in HCV infection. Interestingly, a recent study showed that leptin-induced STAT3 downregulates SREBP-1c expression in hepatic stellate cells (Zhang et al., 2013). Therefore, it appears that STAT3 activity is not strongly associated with GRIM-19 inhibition of SREBP-1c expression.

To date, the role of p53 in lipid metabolism is still uncertain; however, p53 and its target genes may be anti-viral host factors in HCV pathogenesis because they are also impaired by HCV infection (Nishimura et al., 2009). Recently, Zhou et al. (2011) demonstrated that the p53 tumor suppressor was regulated by GRIM-19 expression. They showed that GRIM-19 helps to stabilize p53 by interacting with E6 and E6AP proteins and inducing ubiquitination and degradation of E6AP, resulting in the promotion of apoptosis in a cervical cancer cell line (Zhou et al., 2011). In contrast, another study by Ruedo-Rincon et al. (2015) demonstrated that p53 could decrease SCD expression by repressing SREBP-1c. Based on these results, we speculate that p53 is involved in the negative regulation of SREBP-1c expression by GRIM-19. However, future investigations are needed to understand the exact mechanisms of the interaction between p53 and GRIM-19 during SREBP1-c expression and lipid metabolism.

## Conclusion

Our data reveal a previously unknown role of GRIM-19 in lipid metabolism during HCV pathogenesis. GRIM-19 overexpression abrogated intracellular lipid accumulation through downregulation of SREBP-1c and its target genes, resulting in restriction of HCV replication. These results provide valuable information regarding GRIM-19, a host factor involved in HCV replication. Furthermore, this new knowledge regarding GRIM-19 may facilitate new strategies against diseases related to lipid metabolic disorders.

## Author Contributions

SY, E-CS, and J-HK designed the concepts of this study. J-HK, PS, EL, and DP carried out the experiments. SY, E-CS, J-HK, PS, WH, and MW discussed and interpreted the results. J-HK wrote the manuscripts. SY supervised the experiment and project.

## Conflict of Interest Statement

The authors declare that the research was conducted in the absence of any commercial or financial relationships that could be construed as a potential conflict of interest.

## References

[B1] Ahmadian M. Suh J. M. Hah N. Liddle C. Atkins A. R. Downes M. (2013). PPARgamma signaling and metabolism: the good, the bad and the future. *Nat. Med.* 19 557–566. 10.1038/nm.3159 23652116 PMC3870016

[B2] Aizaki H. Lee K. J. Sung V. M. Ishiko H. Lai M. M. (2004). Characterization of the hepatitis C virus RNA replication complex associated with lipid rafts. *Virology* 324 450–461. 10.1016/j.virol.2004.03.034 15207630

[B3] Akil A. Wedeh G. Zahid Mustafa M. Gassama-Diagne A. (2016). SUMO1 depletion prevents lipid droplet accumulation and HCV replication. *Arch. Virol.* 161 141–148. 10.1007/s00705-015-2628-3 26449956

[B4] Alchanati I. Nallar S. C. Sun P. Gao L. Hu J. Stein A. (2006). A proteomic analysis reveals the loss of expression of the cell death regulatory gene GRIM-19 in human renal cell carcinomas. *Oncogene* 25 7138–7147. 10.1038/sj.onc.1209708 16732315

[B5] Angell J. E. Lindner D. J. Shapiro P. S. Hofmann E. R. Kalvakolanu D. V. (2000). Identification of GRIM-19, a novel cell death-regulatory gene induced by the interferon-beta and retinoic acid combination, using a genetic approach. *J. Biol. Chem.* 275 33416–33426. 10.1074/jbc.M003929200 10924506

[B6] Aslam R. Raza S. M. Naeemi H. Mubarak B. Afzal N. Khaliq S. (2016). SOCS3 mRNA expression and polymorphisms as pretreatment predictor of response to HCV genotype 3a IFN-based treatment. *Springerplus* 5 1826 10.1186/s40064-016-3506-5 PMC5074986 27818864

[B7] Choi J. E. Kwon J. H. Kim J. H. Hur W. Sung P. S. Choi S. W. (2015). Suppression of dual specificity phosphatase I expression inhibits hepatitis C virus replication. *PLoS ONE* 10:e0119172 10.1371/journal.pone.0119172 PMC4370512 25798824

[B8] Date T. Miyamoto M. Kato T. Morikawa K. Murayama A. Akazawa D. (2007). An infectious and selectable full-length replicon system with hepatitis C virus JFH-1 strain. *Hepatol. Res.* 37 433–443. 10.1111/j.1872-034X.2007.00056.x 17437527

[B9] Egger D. Wolk B. Gosert R. Bianchi L. Blum H. E. Moradpour D. (2002). Expression of hepatitis C virus proteins induces distinct membrane alterations including a candidate viral replication complex. *J. Virol.* 76 5974–5984. 10.1128/JVI.76.12.5974-5984.2002 12021330 PMC136238

[B10] El-Saadany S. Ziada D. H. El Bassat H. Farrag W. El-Serogy H. Eid M. (2013). The role of hepatic expression of STAT1, SOCS3 and PIAS1 in the response of chronic hepatitis C patients to therapy. *Can. J. Gastroenterol.* 27 e13–e17. 10.1155/2013/562765 23472246 PMC3731121

[B11] Gale M. Jr. Foy E. M. (2005). Evasion of intracellular host defence by hepatitis C virus. *Nature* 436 939–945. 10.1038/nature04078 16107833

[B12] Garcia-Mediavilla M. V. Pisonero-Vaquero S. Lima-Cabello E. Benedicto I. Majano P. L. Jorquera F. (2012). Liver X receptor alpha-mediated regulation of lipogenesis by core and NS5A proteins contributes to HCV-induced liver steatosis and HCV replication. *Lab. Invest.* 92 1191–1202. 10.1038/labinvest.2012.88 22641099

[B13] George A. Panda S. Kudmulwar D. Chhatbar S. P. Nayak S. C. Krishnan H. H. (2012). Hepatitis C virus NS5A binds to the mRNA cap-binding eukaryotic translation initiation 4F (eIF4F) complex and up-regulates host translation initiation machinery through eIF4E-binding protein 1 inactivation. *J. Biol. Chem.* 287 5042–5058. 10.1074/jbc.M111.308916 22184107 PMC3281608

[B14] Hur W. Kim S. W. Lee Y. K. Choi J. E. Hong S. W. Song M. J. (2012). Oleuropein reduces free fatty acid-induced lipogenesis via lowered extracellular signal-regulated kinase activation in hepatocytes. *Nutr. Res.* 32 778–786. 10.1016/j.nutres.2012.06.017 23146775

[B15] Kalakonda S. Nallar S. C. Lindner D. J. Hu J. Reddy S. P. Kalvakolanu D. V. (2007). Tumor-suppressive activity of the cell death activator GRIM-19 on a constitutively active signal transducer and activator of transcription 3. *Cancer Res.* 67 6212–6220. 10.1158/0008-5472.CAN-07-0031 17616678

[B16] Kalvakolanu D. V. Nallar S. C. Kalakonda S. (2010). Cytokine-induced tumor suppressors: a GRIM story. *Cytokine* 52 128–142. 10.1016/j.cyto.2010.03.009 20382543 PMC2925066

[B17] Kapadia S. B. Chisari F. V. (2005). Hepatitis C virus RNA replication is regulated by host geranylgeranylation and fatty acids. *Proc. Natl. Acad. Sci. U.S.A.* 102 2561–2566. 10.1073/pnas.0409834102 15699349 PMC549027

[B18] Kim S. J. Kim J. H. Sun J. M. Kim M. G. Oh J. W. (2009). Suppression of hepatitis C virus replication by protein kinase C-related kinase 2 inhibitors that block phosphorylation of viral RNA polymerase. *J. Viral. Hepat.* 16 697–704. 10.1111/j.1365-2893.2009.01108.x 19243496

[B19] Kong L. Li S. Yu X. Fang X. Xu A. Huang M. (2016). Hepatitis C virus and its protein NS4B activate the cancer-related STAT3 pathway via the endoplasmic reticulum overload response. *Arch. Virol.* 161 2149–2159. 10.1007/s00705-016-2892-x 27180099

[B20] Li M. Li Z. Liang C. Han C. Huang W. Sun F. (2014). Upregulation of GRIM-19 suppresses the growth of oral squamous cell carcinoma in vitro and in vivo. *Oncol. Rep.* 32 2183–2190. 10.3892/or.2014.3423 25174621

[B21] Li Q. Brass A. L. Ng A. Hu Z. Xavier R. J. Liang T. J. (2009). A genome-wide genetic screen for host factors required for hepatitis C virus propagation. *Proc. Natl. Acad. Sci. U.S.A.* 106 16410–16415. 10.1073/pnas.0907439106 19717417 PMC2752535

[B22] Li Q. Pene V. Krishnamurthy S. Cha H. Liang T. J. (2013). Hepatitis C virus infection activates an innate pathway involving IKK-alpha in lipogenesis and viral assembly. *Nat. Med.* 19 722–729. 10.1038/nm.3190 23708292 PMC3676727

[B23] Li Q. Zhang Y. Y. Chiu S. Hu Z. Lan K. H. Cha H. (2014). Integrative functional genomics of hepatitis C virus infection identifies host dependencies in complete viral replication cycle. *PLoS Pathog.* 10:e1004163 10.1371/journal.ppat.1004163 PMC4095987 24852294

[B24] Licursi M. Komatsu Y. Pongnopparat T. Hirasawa K. (2012). Promotion of viral internal ribosomal entry site-mediated translation under amino acid starvation. *J. Gen. Virol.* 93 951–962. 10.1099/vir.0.040386-0 22302880

[B25] Lindenbach B. D. Rice C. M. (2005). Unravelling hepatitis C virus replication from genome to function. *Nature* 436 933–938. 10.1038/nature04077 16107832

[B26] Liu S. Zhang W. Liu K. Wang Y. Ji B. Liu Y. (2014). Synergistic effects of co-expression plasmidbased ADAM10-specific siRNA and GRIM-19 on hepatocellular carcinoma in vitro and in vivo. *Oncol. Rep.* 32 2501–2510. 10.3892/or.2014.3503 25242535

[B27] Luyimbazi D. Akcakanat A. Mcauliffe P. F. Zhang L. Singh G. Gonzalez-Angulo A. M. (2010). Rapamycin regulates stearoyl CoA desaturase 1 expression in breast cancer. *Mol. Cancer Ther.* 9 2770–2784. 10.1158/1535-7163.MCT-09-0980 20876744 PMC2965451

[B28] Mankouri J. Tedbury P. R. Gretton S. Hughes M. E. Griffin S. D. Dallas M. L. (2010). Enhanced hepatitis C virus genome replication and lipid accumulation mediated by inhibition of AMP-activated protein kinase. *Proc. Natl. Acad. Sci. U.S.A.* 107 11549–11554. 10.1073/pnas.0912426107 20534540 PMC2895084

[B29] McCartney E. M. Helbig K. J. Narayana S. K. Eyre N. S. Aloia A. L. Beard M. R. (2013). Signal transducer and activator of transcription 3 is a proviral host factor for hepatitis C virus. *Hepatology* 58 1558–1568. 10.1002/hep.26496 23703790

[B30] McRae S. Iqbal J. Sarkar-Dutta M. Lane S. Nagaraj A. Ali N. (2015). Hepatitis C virus-induced NLRP3-inflammasome activates the sterol regulatory element binding protein (SREBP) and regulates lipid metabolism. *J. Biol. Chem.* 291 3254–3267. 10.1074/jbc.M115.694059 26698881 PMC4751372

[B31] Memon R. A. Tecott L. H. Nonogaki K. Beigneux A. Moser A. H. Grunfeld C. (2000). Up-regulation of peroxisome proliferator-activated receptors (PPAR-alpha) and PPAR-gamma messenger ribonucleic acid expression in the liver in murine obesity: troglitazone induces expression of PPAR-gamma-responsive adipose tissue-specific genes in the liver of obese diabetic mice. *Endocrinology* 141 4021–4031. 10.1210/endo.141.11.7771 11089532

[B32] Meredith L. W. Wilson G. K. Fletcher N. F. Mckeating J. A. (2012). Hepatitis C virus entry: beyond receptors. *Rev. Med. Virol.* 22 182–193. 10.1002/rmv.723 22392805

[B33] Miyanari Y. Atsuzawa K. Usuda N. Watashi K. Hishiki T. Zayas M. (2007). The lipid droplet is an important organelle for hepatitis C virus production. *Nat. Cell Biol.* 9 1089–1097. 10.1038/ncb1631 17721513

[B34] Moreira S. Correia M. Soares P. Maximo V. (2011). GRIM-19 function in cancer development. *Mitochondrion* 11 693–699. 10.1016/j.mito.2011.05.011 21664299

[B35] Nallar S. C. Kalakonda S. Sun P. Kalvakolanu D. V. (2008). GRIM-19: a double-edged sword that regulates anti-tumor and innate immune responses. *Transl. Oncogenomics* 3 67–79. 21566745 PMC3022361

[B36] Negro F. Sanyal A. J. (2009). Hepatitis C virus, steatosis and lipid abnormalities: clinical and pathogenic data. *Liver Int.* 29(Suppl. 2), 26–37. 10.1111/j.1478-3231.2008.01950.x 19187070

[B37] Nishimura T. Kohara M. Izumi K. Kasama Y. Hirata Y. Huang Y. (2009). Hepatitis C virus impairs p53 via persistent overexpression of 3beta-hydroxysterol Delta24-reductase. *J. Biol. Chem.* 284 36442–36452. 10.1074/jbc.M109.043232 19861417 PMC2794760

[B38] Panda S. Vedagiri D. Viveka T. S. Harshan K. H. (2014). A unique phosphorylation-dependent eIF4E assembly on 40S ribosomes co-ordinated by hepatitis C virus protein NS5A that activates internal ribosome entry site translation. *Biochem. J.* 462 291–302. 10.1042/BJ20131530 24894874

[B39] Park C. Y. Jun H. J. Wakita T. Cheong J. H. Hwang S. B. (2009). Hepatitis C virus nonstructural 4B protein modulates sterol regulatory element-binding protein signaling via the AKT pathway. *J. Biol. Chem.* 284 9237–9246. 10.1074/jbc.M808773200 19204002 PMC2666576

[B40] Pisonero-Vaquero S. Garcia-Mediavilla M. V. Jorquera F. Majano P. L. Benet M. Jover R. (2014). Modulation of PI3K-LXRalpha-dependent lipogenesis mediated by oxidative/nitrosative stress contributes to inhibition of HCV replication by quercetin. *Lab. Invest.* 94 262–274. 10.1038/labinvest.2013.156 24492281

[B41] Reeves M. B. Davies A. A. Mcsharry B. P. Wilkinson G. W. Sinclair J. H. (2007). Complex I binding by a virally encoded RNA regulates mitochondria-induced cell death. *Science* 316 1345–1348. 10.1126/science.1142984 17540903

[B42] Ruedo-Rincon N. Bloch K. Derua R. Vyas R. Harms A. Hankemeier T. (2015). p53 attenuates AKT signaling by modulating membrane phospholipid composition. *Oncotarget* 6 21240–21254. 10.18632/oncotarget.4067 26061814 PMC4673262

[B43] Sanders F. W. Griffin J. L. (2016). De novo lipogenesis in the liver in health and disease: more than just a shunting yard for glucose. *Biol. Rev. Camb. Philos. Soc.* 91 452–468. 10.1111/brv.12178 25740151 PMC4832395

[B44] Seo T. Lee D. Shim Y. S. Angell J. E. Chidambaram N. V. Kalvakolanu D. V. (2002). Viral interferon regulatory factor 1 of Kaposi’s sarcoma-associated herpesvirus interacts with a cell death regulator, GRIM19, and inhibits interferon/retinoic acid-induced cell death. *J. Virol.* 76 8797–8807. 10.1128/JVI.76.17.8797-8807.2002 12163600 PMC136415

[B45] Shulga N. Pastorino J. G. (2012). GRIM-19-mediated translocation of STAT3 to mitochondria is necessary for TNF-induced necroptosis. *J. Cell Sci.* 125 2995–3003. 10.1242/jcs.103093 22393233 PMC3434811

[B46] Su A. I. Pezacki J. P. Wodicka L. Brideau A. D. Supekova L. Thimme R. (2002). Genomic analysis of the host response to hepatitis C virus infection. *Proc. Natl. Acad. Sci. U.S.A.* 99 15669–15674. 10.1073/pnas.202608199 12441396 PMC137774

[B47] Sun J. M. Kim S. J. Kim G. W. Rhee J. K. Kim N. D. Jung H. (2012). Inhibition of hepatitis C virus replication by Monascus pigment derivatives that interfere with viral RNA polymerase activity and the mevalonate biosynthesis pathway. *J. Antimicrob. Chemother.* 67 49–58. 10.1093/jac/dkr432 22076990 PMC7109977

[B48] Sung P. S. Cheon H. Cho C. H. Hong S. H. Park D. Y. Seo H. I. (2015). Roles of unphosphorylated ISGF3 in HCV infection and interferon responsiveness. *Proc. Natl. Acad. Sci. U.S.A.* 112 10443–10448. 10.1073/pnas.1513341112 26216956 PMC4547285

[B49] Suzuki T. (2012). Morphogenesis of infectious hepatitis C virus particles. *Front. Microbiol.* 3:38 10.3389/fmicb.2012.00038 PMC3273859 22347224

[B50] Syed G. H. Amako Y. Siddiqui A. (2010). Hepatitis C virus hijacks host lipid metabolism. *Trends Endocrinol. Metab.* 21 33–40. 10.1016/j.tem.2009.07.005 19854061 PMC2818172

[B51] Vallianou I. Dafou D. Vassilaki N. Mavromara P. Hadzopoulou-Cladaras M. (2016). Hepatitis C virus suppresses hepatocyte nuclear factor 4 alpha, a key regulator of hepatocellular carcinoma. *Int. J. Biochem. Cell Biol.* 78 315–326. 10.1016/j.biocel.2016.07.027 27477312

[B52] Wakita T. Pietschmann T. Kato T. Date T. Miyamoto M. Zhao Z. (2005). Production of infectious hepatitis C virus in tissue culture from a cloned viral genome. *Nat. Med.* 11 791–796. 10.1038/nm1268 15951748 PMC2918402

[B53] Waris G. Felmlee D. J. Negro F. Siddiqui A. (2007). Hepatitis C virus induces proteolytic cleavage of sterol regulatory element binding proteins and stimulates their phosphorylation via oxidative stress. *J. Virol.* 81 8122–8130. 10.1128/JVI.00125-07 17507484 PMC1951320

[B54] Xiang Z. Qiao L. Zhou Y. Babiuk L. A. Liu Q. (2010). Hepatitis C virus nonstructural protein-5A activates sterol regulatory element-binding protein-1c through transcription factor Sp1. *Biochem. Biophys. Res. Commun.* 402 549–553. 10.1016/j.bbrc.2010.10.081 20971080

[B55] Xu G. Yang F. Ding C. L. Wang J. Zhao P. Wang W. (2014). MiR-221 accentuates IFNs anti-HCV effect by downregulating SOCS1 and SOCS3. *Virology* 46 343–350. 10.1016/j.virol.2014.06.024 25019494

[B56] Yang W. Hood B. L. Chadwick S. L. Liu S. Watkins S. C. Luo G. (2008). Fatty acid synthase is up-regulated during hepatitis C virus infection and regulates hepatitis C virus entry and production. *Hepatology* 48 1396–1403. 10.1002/hep.22508 18830996 PMC2614928

[B57] Yoshida T. Hanada T. Tokuhisa T. Kosai K. Sata M. Kohara M. (2002). Activation of STAT3 by the hepatitis C virus core protein leads to cellular transformation. *J. Exp. Med.* 196 641–653. 10.1084/jem.20012127 12208879 PMC2194001

[B58] Zhang W. Niu M. Yan K. Zhai X. Zhou Q. Zhang L. (2013). Stat3 pathway correlates with the roles of leptin in mouse liver fibrosis and sterol regulatory element binding protein-1c expression of rat hepatic stellate cells. *Int. J. Biochem. Cell Biol.* 45 736–744. 10.1016/j.biocel.2012.12.019 23295202

[B59] Zhao L. J. He S. F. Wang W. Ren H. Qi Z. T. (2016). Interferon alpha antagonizes STAT3 and SOCS3 signaling triggered by hepatitis C virus. *Cytokine* 80 48–55. 10.1016/j.cyto.2015.08.264 26945996

[B60] Zhou Y. Wei Y. Zhu J. Wang Q. Bao L. Ma Y. (2011). GRIM-19 disrupts E6/E6AP complex to rescue p53 and induce apoptosis in cervical cancers. *PLoS ONE* 6:e22065 10.1371/journal.pone.0022065 PMC3134474 21765936

